# Identification of Promising Mutants Associated with Egg Production Traits Revealed by Genome-Wide Association Study

**DOI:** 10.1371/journal.pone.0140615

**Published:** 2015-10-23

**Authors:** Jingwei Yuan, Congjiao Sun, Taocun Dou, Guoqiang Yi, LuJiang Qu, Liang Qu, Kehua Wang, Ning Yang

**Affiliations:** 1 National Engineering Laboratory for Animal Breeding and MOA Key Laboratory of Animal Genetics and Breeding, College of Animal Science and Technology, China Agricultural University, Beijing, 100193, P.R. China; 2 Jiangsu Institute of Poultry Science, Yangzhou, 225125, P.R. China; Chinese Academy of Fishery Sciences, CHINA

## Abstract

Egg number (EN), egg laying rate (LR) and age at first egg (AFE) are important production traits related to egg production in poultry industry. To better understand the knowledge of genetic architecture of dynamic EN during the whole laying cycle and provide the precise positions of associated variants for EN, LR and AFE, laying records from 21 to 72 weeks of age were collected individually for 1,534 F_2_ hens produced by reciprocal crosses between White Leghorn and Dongxiang Blue-shelled chicken, and their genotypes were assayed by chicken 600 K Affymetrix high density genotyping arrays. Subsequently, pedigree and SNP-based genetic parameters were estimated and a genome-wide association study (GWAS) was conducted on EN, LR and AFE. The heritability estimates were similar between pedigree and SNP-based estimates varying from 0.17 to 0.36. In the GWA analysis, we identified nine genome-wide significant loci associated with EN of the laying periods from 21 to 26 weeks, 27 to 36 weeks and 37 to 72 weeks. Analysis of *GTF2A1* and *CLSPN* suggested that they influenced the function of ovary and uterus, and may be considered as relevant candidates. The identified SNP *rs314448799* for accumulative EN from 21 to 40 weeks on chromosome 5 created phenotypic differences of 6.86 eggs between two homozygous genotypes, which could be potentially applied to the molecular breeding for EN selection. Moreover, our finding showed that LR was a moderate polygenic trait. The suggestive significant region on chromosome 16 for AFE suggested the relationship between sex maturity and immune in the current population. The present study comprehensively evaluates the role of genetic variants in the development of egg laying. The findings will be helpful to investigation of causative genes function and future marker-assisted selection and genomic selection in chickens.

## Introduction

Egg production traits, including egg number, egg mass and egg laying rate, have always been a focus of attention in the poultry breeding. Egg number (EN) and egg laying rate as the most meaningful traits in layers breeding program has procured considerable genetic progress in commercial egg-layer breeds through traditional selection for several decades, reaching a level at an egg on almost every day in highly efficient hens [1]. Similarly, age at first egg (AFE) was also an important indicator for egg production performance. Nowadays, young hens as early as 17 wks of age start to produce the first egg. With the development of high-throughput genotyping platforms, the genetic gain of egg production traits can be still increased by using new molecular breeding strategy, especially for the indigenous chickens in the developing countries.

Genetic variations in egg production traits can be dissected and quantified with the associated genetic markers. Thus, to identify genetic variants affecting egg production traits is one of primary goals in the poultry genetics for more than fifteen years. Numerous previous studies had been conducted to map or identify QTLs and SNPs that associated with EN. However, most of these candidates were cross-sectional in a specific laying period [2–5], which had relatively poor power to unravel the genetic control of EN in the whole laying cycle. In addition, the joint use of phenotypic, genomic and pedigree information for selection brought a new impetus into EN breeding of chickens [6]. Therefore, it is necessary for the further investigation for the genetic architectures of EN in a more comprehensive perspective. Exactly, egg production performance of various laying periods and a higher density genotyping array for chicken genome should be combined to study the genetic architectures of dynamic EN. Moreover, Chinese indigenous breed, which is advantageous in egg quality and flavor but inferior to the commercial breeds in egg production, need an imperative improvement of egg production traits.

An F_2_ cross population design has obtained success in QTL mapping studies and GWA studies for target traits in chicken [7, 8]. Herein, two breeds with markedly physiological, morphological and production differences are chosen for the reciprocal crosses to produce the F_2_ offspring. Of the two breeds, White Leghorn (WL) is a world-wide standard layer breed with high egg production performance, while Dongxiang Blue-shelled (DBS) chicken is a Chinese local breed with relatively low laying performance [9]. Meanwhile, the application of the chicken 600 K SNP array [10] allows genotyping at a higher marker density and revealing previously undetected associations and precise locations of variants. Therefore, on the basis of F_2_ design population and chicken 600 K SNP arrays, the current study aim to identify the patterns of genetic control for EN at various laying period from 21 to 72 weeks of age and to unveil possible mutants and genes of interest. In addition, we expect to provide some promising candidate genes for egg laying rate and age at first egg.

## Materials and Methods

### Ethics Statement

Blood samples were collected from brachial veins of chickens by standard venipuncture along with the regular quarantine inspection of the experimental station of China Agricultural University in accordance with *the Guidelines for the Care and Use of Experimental Animals* established by the Ministry of Agriculture of China (Beijing, China). The entire study was approved by the Animal Welfare Committee of China Agricultural University (permit number: SYXK 2007–0023).

### Population and Trait Measurements

A chicken F_2_ resource population was derived from reciprocal cross between two breeds that differed in egg production, i.e. White Leghorn (WL) originated from Shanghai Poultry Breeding Co., Ltd with selection on egg production and quality, and Dongxiang Blue-shelled chicken (DBS) which has been selected for egg production and egg-shell color since 1998 at the experimental farm in Jiangsu Institute of Poultry Science. Briefly, reciprocal mating of unrelated 6 WL(♂) × 133 DBS (♀) and unrelated 6 DBS(♂) × 80 WL (♀) was used to produce the F_1_ generation. Unrelated F_1_ chickens, involving 25 males and 406 females from WL/DBS pair and 24 males and 233 females from DBS/WL pair, were randomly selected to produce the F_2_ generation. In F_2_, a total of 3,749 F_2_ birds including 1,856 males and 1,893 females were produced from 590 half-sib families in a single hatch as described previously [11]. Hens were housed in individual cages in 2 identical houses under the standard management conditions at the same feedlot at the research station of Jiangsu Institute of Poultry Science. Each bird was provided ad libitum access to water and a commercial corn–soybean diet that met National Research Council (NRC) requirements during the study period.

At 17 weeks of age, birds were moved to the laying house and kept in the individual stair-step cages for one week adaption. For each bird, age at the first egg (AFE) was recorded. The number of eggs produced from AFE to 72 wks of age was daily recorded for each bird, and then egg numbers were divided into five parts based on the characterization of egg production curve (S1 Fig), including pre-peak laying stage from 21 to 26 weeks (EN1), peak laying stage from 27 to 36 weeks (EN2) and persistent laying stage 37 to 72 weeks (EN3). Of the three laying stages, EN3 was further divided into EN4 from 37 to 47 weeks (70% ≤ laying rate < 80%) and EN5 from 48 to 72 weeks (laying rate < 70%) based on the egg laying rate. Accumulative egg number from 21 to 40 weeks (EN21-40), to 56 weeks (EN21-56) and to 72 weeks (EN21-72) were collected for available hens. In addition, egg laying rate (LR) was calculated as (the number of eggs) / (the laying days between 25 and 40 weeks of age) multiplied by 100% [3]. Hens with an egg production < 109 (< 30% laying rate) in the whole laying cycle were excluded for further analysis [12]. For each trait, phenotypic values that did not fall into the range of [mean ±3 standard deviations (SDs)] were removed prior to the analysis.

### Genotyping and Imputation

Genomic DNA was isolated from whole blood samples using phenol-chloroform methods. A total of 1,534 F_2_ hens were genotyped for 580,961 markers using Affymetrix 600 K chicken high density genotyping array [10] completed by GeneSeek, Inc (Lincoln, NE, USA). We assessed reproducibility by genotyping 2 samples in duplicate, and 99.8% identical genotype calls were observed. In the quality control, twenty-two samples were eliminated with a missing SNP call rate >5% using Affymetrix power tool (APT) provided by Affymetrix (http://affymetrix.com/) and the final average sample call rate was 99.2%. And then all autosomal SNPs from 1,512 qualified samples that met quality control criteria that set in PLINK [13] (>95% call rate, >1% minor allele frequencies and Hardy Weinberg equilibrium *P*-value < 1e-6) were used for imputation implemented in Beagle Version 4 software package based on localized haplotype clustering [14]. Finally, a total of 435,243 SNPs and 1,512 birds were obtained for the further analyses after filtering for imputation results using PLINK.

### Whole genome association studies

Subsequently, the eligible SNPs and birds were used to evaluate the population structure by PLINK [13]. Firstly, all SNPs were pruned to obtain independent SNP markers using the indep-pairwise option, with a window size of 25 SNPs, a step of 5 SNPs, and r^2^ threshold of 0.2. Secondly, pairwise identity-by-state (IBS) distances were calculated between all individuals using the independent SNP markers. Finally, we calculated multidimensional scaling (MDS) components using the mds-plot option based on the IBS matrix, which was included as covariate in the subsequent GWAS analyses [15].

Genome-wide association study analysis was performed using mixed models approach [16] implemented in GEMMA software. The package fitted a linear mixed model to account for population stratification and sample structure with a faster computational time for thousands of individuals [17]. Association test with univariate linear mixed model (univariate GWAS) was performed for each trait. The statistical model was:

y=Wα+xβ+u+ε (1)

where *y* is the vector of traits value for all individuals; **W** is an matrix of covariates (fixed effects contain first MDS component and a column of 1s); **α** is a vector of the corresponding coefficients including the intercept; **x** is an vector of marker genotypes; *β* is the effect size of the marker; **u** is vector of individual random effects; **ε** is vector of errors. Wald test statistic was used as criteria to screen SNPs significantly associated with the investigated traits.

With respect to the genome-wide significant *P*-value threshold, simple**M** method [18] was used to infer the independent test, resulting in 59,286 independent tests over the entire autosomal SNPs, and then genome-wise significance and suggestive significance were calculated as 8.43×10^−7^ (0.05/59,286) and 1.69×10^−5^ (1.00/59,286), respectively. Similarly, the chromosome-wide significant *P*-value threshold was adjusted based on the independent tests in each chromosome. The Manhattan and Q-Q plot were constructed for each trait by the GAP package (http://cran.r-project.org/web/packages/gap/index.html) within the R software [19].

### Post GWA analysis

Linkage disequilibrium (LD) analysis were performed for the chromosomal regions with many significant SNPs clustered using software Haploview version 4.2 [20] with algorithm proposed by Gabriel et al. [21]. A further association analysis were conducted for the identified LD blocks completed with *haplo*.*score()* in the Haplo.Stats R-package, which calculated score statistics to evaluate the association of a trait with haplotype for ambiguous linkage phase [22]. Of the score statistics, a globe *P*-value was calculated to test overall associations among LD blocks and traits, and *P*-value for each haplotype was calculated to test significance between haplotype and traits. Score (Hap-Score) and frequency (Hap-Freq) for a particular haplotype were also provided in the results.

Pedigree-based genetic parameters for egg production were estimated with the univariate and bivariate (two-trait) animal model implemented in DMU software [23] as follow:

y=1μ+Za+e (2)

where y is the phenotypic value of the trait, 1 and Z are the incidence matrix of fixed effects (population means) and random effects (individual additive genetic effect), respectively, μ and a are the vectors of fixed effects and random additive effects, respectively, e is the random residual effect. The pedigree structure contained 12 sires and 213 dams from the parent generation, 49 males and 639 females from the F_1_ generation, and available hens from the F_2_ generation. Among these animals, only F_2_ birds were phenotyped for egg production traits. On the other hand, estimation of the phenotypic variance explained by significantly associated SNPs and all SNPs (SNP-based heritability [24] and genetic correlation [25]) were calculated by Restricted Maximum Likelihood (REML) analysis using GCTA software [26].

SNP positions and information were obtained using annotation of *Gallus gallus* 4.0 genome version, and genes within 500,000 base pairs flanking the associated SNPs were chose for the further analysis. The positional annotation genes were extract from NCBI database using *GetNeighGenes()* in the NCBI2R R-package (http://cran.r-project.org/web/packages/NCBI2R/index.html). Investigation of gene ontology (GO) and the relevant Kyoto Encyclopedia of Genes and Genomes (KEGG) pathways for the genes within 1Mb of associated SNPs was performed to determine biological processes and pathways associated with traits using the Database for Annotation, Visualization and Integrated Discovery (DAVID) [27].

## Results

The detailed information for all autosomal SNPs that passed the quality control and independent test for 28 autosomes and 2 linkage groups are respectively shown in Table 1. Descriptive statistics of egg production traits for genotyped individuals that pass the quality control are listed in Table 2.

**Table 1:** Basic information for SNP markers on a physical map after quality control.

Chromosome	Map distance (Kb)^1^	No. SNPs	Density (kb/SNP)	Inferred_Meff ^2^	Chromosome	Map distance (Kb)	No. SNPs	Density (kb/SNP)	Inferred_Meff
1	195235.1	81104	2.4	10322	16	492.2	323	1.5	57
2	148789.3	52713	2.8	7139	17	10280.4	7541	1.4	1153
3	110439.9	45712	2.4	6053	18	11198.7	7665	1.5	1147
4	90168.3	35747	2.5	5132	19	9982.0	6979	1.4	902
5	59545.2	24659	2.4	3441	20	14274.8	7525	1.9	1037
6	34945.8	17886	2.0	2334	21	6785.5	6800	1.0	837
7	36195.7	17649	2.1	2269	22	4075.8	3314	1.2	508
8	28744.6	13870	2.1	1847	23	5707.8	5019	1.1	873
9	23422.5	14612	1.6	1942	24	6312.9	5922	1.1	846
10	19904.7	14956	1.3	1852	25	2170.8	1767	1.2	343
11	19381.0	11253	1.7	1506	26	5320.3	4573	1.2	770
12	19850.5	11420	1.7	1564	27	5194.8	3997	1.3	707
13	17755.0	9074	2.0	1281	28	4735.8	4032	1.2	645
14	15145.4	10528	1.4	1415	LGE64	961.2	139	6.9	66
15	12644.7	8415	1.5	1120	LGE22^3^	648.0	49	13.2	15
Total	920308.7	435243		59286					

^1^The physical length of the chromosome was based on the position of the last marker in the *Gullus gullus* version 4

^2^ Inferred_Meff, effective number of independent tests

^3^LGE22, linkage group LGE22C19W28_E50C23.

**Table 2:** Descriptive statistics of egg production traits for F_2_ chickens.

Traits^1^	n^2^	Mean	SD	CV (%)^3^	Min	Max
EN1	1452	22.65	8.15	36.00	1	41
EN2	1457	57.18	6.49	11.35	34	70
EN3	1473	163.12	29.49	18.08	73	224
EN4	1455	57.51	7.70	13.39	31	76
EN5	1469	106.28	23.59	22.20	35	154
EN21-40	1489	100.74	14.64	14.36	52	133
EN21-56	1461	176.35	23.56	13.36	100	226
EN21-72	1494	242.37	36.33	14.99	128	316
LR	1489	79.28%	9.14	11.53	46.43%	100.00%
AFE	1530	153.47	10.73	6.99	133	203

^1^: EN1, egg number in the pre-peak laying period from 21 to 26 weeks of age; EN2, egg number in the peak laying period from 27 to 36 weeks of age; EN3, egg number in the persistent laying period from 37 to 72 weeks of age; EN4, egg number from 37 to 47 weeks of age; EN5, egg number from 48 to 72 weeks of age; EN21-40, egg number from 21 to 40 weeks of age; EN21-56, egg number from 21 weeks of age to 56 weeks of age; EN21-72, egg number from 21 to 72 weeks of age; LR, egg laying rate from 25 to 40 weeks of age; AFE, age at first egg.

^2^: Number of birds that pass the quality control of phenotypic value.

^3^: Coefficient of variation.

### Genetic parameter estimates

Genetic parameters of egg number (EN1-5), egg laying rate (LR) and age at first egg (AFE) are presented in Table 3. Similar heritability estimates between pedigree and SNP-based data were found for each trait. The pedigree-based heritability estimates for egg number were higher in the early laying period (EN1-2) than in the late laying period (EN3-5) varying from 0.17 to 0.29, and the highest heritability (0.29) was found for EN2. The SNP-based heritability estimates for egg number were comparable to the pedigree-based heritability estimates ranging from 0.17 to 0.32 during the five laying periods, while the highest heritability estimate was 0.32 for EN1. With respect to the genetic correlations among EN1-5, much lower SNP-based genetic correlations were found between the EN1 and EN3-5 (0.12–0.33) than that of pedigree-based genetic correlations (0.48–0.86). The SNP-based genetic correlations between EN2 and EN3-5 were medium to high (0.54–0.96), and slightly higher correlations were obtained by using pedigree (0.68–1.00). Moreover, LR was positively correlated with egg number in each stage at a high level, varying from 0.59 to 0.99, while AFE showed a negative or low correlations with other traits (-0.97–0.05) in both SNP-based and pedigree-based estimates.

**Table 3:** Genetic parameters of egg number, egg laying rate and age at first egg^1^.

Traits^2^	EN1		EN2		EN3		EN4		EN5		LR		AFE	
EN1	**0.32 (0.04)**	***0*.*26 (0*.*06)***	0.60 (0.10)		0.18 (0.13)		0.33 (0.13)		0.12 (0.13)		0.59 (0.09)		NC^3^	
EN2	*0*.*85 (0*.*09)*		**0.20 (0.04)**	***0*.*29 (0*.*06)***	0.65 (0.10)		0.96 (0.05)		0.54 (0.12)		0.99 (0.01)		-0.43 (0.11)	
EN3	*0*.*64 (0*.*15)*		*0*.*80 (0*.*09)*		**0.20 (0.04)**	***0*.*20 (0*.*05)***	0.89 (0.04)		0.99 (0.01)		0.70 (0.09)		-0.02 (0.12)	
EN4	*0*.*86 (0*.*11)*		*1*.*00 (0*.*03)*		*0*.*90 (0*.*15)*		**0.18 (0.04)**	***0*.*19 (0*.*06)***	0.81 (0.08)		0.96 (0.04)		-0.16 (0.13)	
EN5	*0*.*48 (0*.*18)*		*0*.*68 (0*.*13)*		*0*.*98 (0*.*01)*		*0*.*82 (0*.*09)*		**0.17 (0.04)**	***0*.*17 (0*.*05)***	0.59 (0.11)		0.05 (0.13)	
LR	*0*.*84 (0*.*09)*		*0*.*99 (0*.*004)*		*0*.*83 (0*.*08)*		*1*.*00 (0*.*02)*		*0*.*70 (0*.*12)*		**0.21 (0.04)**	***0*.*29 (0*.*06)***	-0.38 (0.11)	
AFE	*-0*.*97 (0*.*02)*		*-0*.*65 (0*.*13)*		*-0*.*30 (0*.*19)*		*-0*.*55 (0*.*17)*		*-0*.*18 (0*.*20)*		*-0*.*61 (0*.*14)*		**0.36 (0.04)**	***0*.*27 (0*.*07)***

^1^: Heritability is given on diagonal (bold is SNP-based and italic bold is pedigree-based heritability), SNP-based genetic correlations above diagonal and pedigree-based genetic correlations below diagonal. Standard errors of estimates are in parentheses.

^2^: EN1, egg production from 21 to 26 weeks of age; EN2, egg production from 27 to 36 weeks of age; EN3, egg production from 37 to 72 weeks of age; EN4, egg production from 37 to 47 weeks of age; EN5, egg production from 48 to 72 weeks of age; LR, egg laying rate from 25 to 40 weeks of age; AFE, age at first egg.

^3^: NC indicates that the model would not converge.

### Loci identified by GWA analysis

#### Egg number

**Fig 1:**
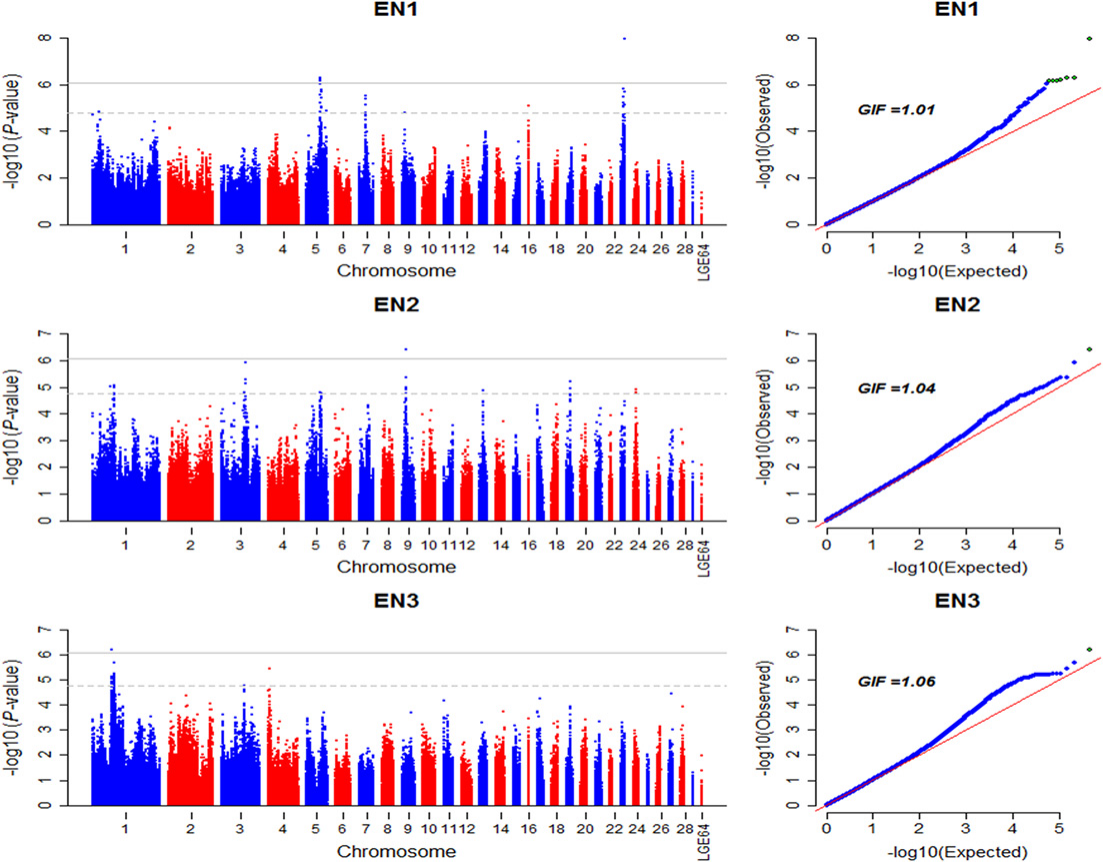
Manhattan and Q-Q plot of genome wide association study for egg number. Each dot represents a SNP in the dataset. Manhattan plot (left). EN1, egg numbers in pre-peak laying stage from 21 to 26 weeks of age. SNPs showing association with EN1 are mapped to one signal in chromosome 5 and a singleton in chromosome 23; EN2, egg numbers in peak laying stage from 27 to 36 weeks of age. SNPs showing association with EN2 are mapped to a singleton in chromosome 9; EN3, egg numbers in persistent laying stage from 37 to 72 weeks of age. SNPs showing association with EN3 are mapped to a singleton in chromosome 1. The horizontal gray line and gray dashed line indicate the genome-wise significance threshold (*P*-value = 8.43e-7) and genome-wise suggestive significance threshold (*P*-value = 1.69e-5), respectively. GIF represents genomic inflation factor.

The Manhattan and Q-Q plot for egg number in the pre-peak, peak and persistent laying stages (EN1-3) are shown in Fig 1. Characterization of markers significantly associated with egg number is summarized in Table 4.

**Table 4:** The information for SNPs significantly associated with egg number in the five laying stages.

Traits^1^	SNP	GGA^2^	Position (bp)	Alleles^3^	MAF^4^	*P*-value	Effect ± SE^5^	Candidate/Nearest genes	Location (kb)^6^
**EN1**	rs317410777	23	4,191,027	G/A	0.47	1.13E-08	1.91±0.33	*CLSPN*	D 1.02
	rs317449530	5	40,101,576	A/G	0.31	4.93E-07	2.04±0.40	*GTF2A1*	3’ UTR
	rs313187645	5	40,106,943	A/G	0.31	4.93E-07	2.04±0.40	*GTF2A1*	Intron 7
	rs312299419	5	40,167,320	G/T	0.28	6.28E-07	2.12±0.42	*STON2*	Intron 4
	rs315655133	5	40,151,832	T/C	0.28	7.14E-07	2.12±0.43	*STON2*	Exon 5
	rs315876467	5	40,178,369	C/T	0.28	7.14E-07	2.12±0.43	*STON2*	Intron 4
	rs315696308	5	40,181,134	A/G	0.28	7.14E-07	2.12±0.43	*STON2*	Intron 4
**EN2**	rs317773842	9	7,473,958	A/G	0.10	3.81E-07	-2.25±0.44	*FARSB*	3’ UTR
**EN3**	rs312387499	1	56,459,390	G/A	0.49	6.50E-07	-6.20±1.24	*KIAA1549*	Intron 18

^1^: EN1, EN2 and EN3 represent egg numbers from 21 to 26 weeks of age, from 27 to 36 weeks of age and from 37 to 72 weeks of age, respectively.

^2^: Chicken chromosome.

^3^: first listed marker is minor allele.

^4^: minor allele frequency.

^5^: allele substitution effect.

^6^: D indicates that the SNP is in the downstream of the gene; UTR indicates untranslated region.

**Fig 2:**
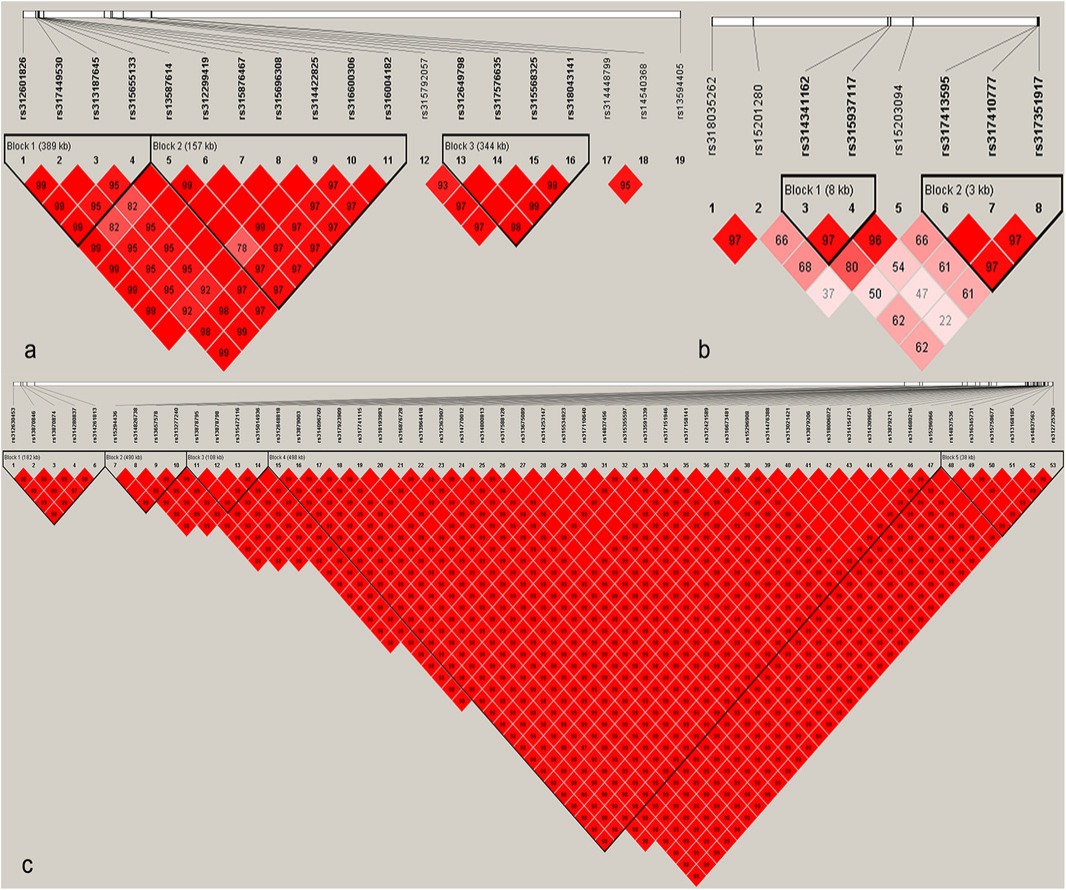
Linkage disequilibrium (r^2^) plot of associations (*P*-value < 1.69e-5) with egg number. (a) and (b) indicate haplotype block on GGA5 and GGA23 for markers showing associations with egg number in the pre-peak laying period, respectively. Haplotype block on for markers showing associations with egg number in the pre-peak laying period. (c) indicates haplotype block on GGA1 for markers showing associations with egg number in the persistent laying period. Solid lines mark the identified blocks.

In the pre-peak laying stage (21–26 weeks), six loci located on genomic region spanning from 39.76 to 43.16 Mb on chromosome 5 (GGA5) significantly associated with egg number, which together explained 2.00% (SE = 0.02) of phenotypic variance. Linkage disequilibrium (LD) analysis for 19 SNPs that passed suggestive significant threshold (*P*-value = 1.69e-5) in this region showed that 15 of these SNPs were clustered in three blocks with scale of 389 kb, 157 kb and 334 kb, respectively (Fig 2A). Association analysis of these three blocks found that haplotype GGGC (0.68, with a negative effect) and AAAT (0.27, with a positive effect) in block 1 were the most significantly associated haplotypes (*P*-value < 1e-5, Table 5) for egg number. The candidate genes harboring or near to the significant SNPs involved in two genes, including *general transcription factor IIA*, *1*, *19/37kDa* (*GTF2A1*) and *stonin 2* (*STON2*). It was notable that *STON2* was an important paralog of *GTF2A1*. In addition to associations on GGA5, three associated genomic regions were located on GGA7, GGA16 and GGA23, respectively. The most significant SNP (*rs317410777*, *P*-value = 1.13e-8), explaining 2.61% (SE = 0.04) of phenotypic variance, situated in a 3 kb block on GGA23 (Fig 2B). The nearest gene to the SNP was *claspin* (*CLSPN*) locating in the upstream 1.02 kb of the SNP at 4.19 Mb on GGA23.

**Table 5:** Results of haplotype association analysis for linkage disequilibrium blocks showing association with egg number from 21 to 26 weeks on GGA5.

Hap	Globe *P*-value^1^	Haplotype	Hap-Freq^2^	Hap-Score^3^	Haplotype-Specific *P*-value^4^
Block1	1e-5	GGGC	0.68	-5.32	<1e-5
		AAAT	0.27	4.85	<1e-5
Block2	3e-4	GTTGGAA	0.60	-4.15	3e-5
		AGCAAGG	0.27	4.95	<1e-5
Block3	4e-5	CATC	0.67	-5.04	<1e-5
		TGCT	0.29	4.61	<1e-5

^1^: The overall association between haplotype and the trait

^2^: Estimated frequency of each haplotype in the population

^3^: The score for the haplotype, which is the statistical measurement of association of each specific haplotype with the trait, and a positive/negative score for a haplotype suggests that the haplotype is associated with increased/decreased trait value

^4^: The asymptotic chi-square *P*-value was calculated from the square of the score statistic.

We extracted 350 nearby genes within 1 Mb of SNPs that surpassed the suggestive significant threshold (*P*-value = 1.69e-5) from NCBI database. These genes were used to perform gene ontology (GO) based on biological process and KEGG pathway analysis in DAVID (available at http://david.abcc.ncifcrf.gov/home.jsp). Twelve significant GO terms and two KEGG pathways were identified (S1 and S2 Tables), and the most significant GO terms was related to antigen processing and presentation and immune response, suggesting that biologically immunologic process may relate to hen production in the pre-peak period in the current population. Significant KEGG pathways included cell adhesion molecules and MAPK signaling pathway.

In GWA analysis of egg number in the peak laying period (EN2), one genome-wide significance (*P*-value < 8.43e-7) and three genome-wide suggestive significance (*P*-value < 1.69e-5) SNPs in an genomic region from 7.47 to 7.54 Mb on GGA9 were related to egg number with negative effects for minor alleles. The most significant SNP, locating in 3’-UTR of *phenylalanyl-tRNA synthetase*, *beta subunit* (*FARSB)*, explained 2.24% (SE = 0.03) of phenotypic variance. In addition to the associated hits on GGA9, five suggestive significant associations were situated on GGA1, GGA3, GGA5, GGA19 and GGA24. The positions and annotations for associated SNPs are provided in S3 Table.

A signal peak on GGA1 from 56.34 Mb to 65.16 Mb was identified to be associated with egg number in the persistent laying period (EN3). These SNPs were in strong LD status (D’ ≥ 0.98, Fig 2C), together explaining 4.56% (SE = 0.04) of phenotypic variance. One SNP in the block 1, locating in the 18^th^ intron of *KIAA1549* gene, was significantly associated with EN3. However, no genome-wide significant association was detected in the GWA analysis of the EN4 and EN5 that derived from EN3 based on the laying rate, and associations in the two laying periods were different from each other (S2 Fig). GWA analysis for EN5 identified more suggestive associations comparing to EN4. Four suggestive significant SNPs in a sharp region on GGA4 were identified for EN5. The characterizations of associated SNPs are provided in S3 Table.

**Fig 3:**
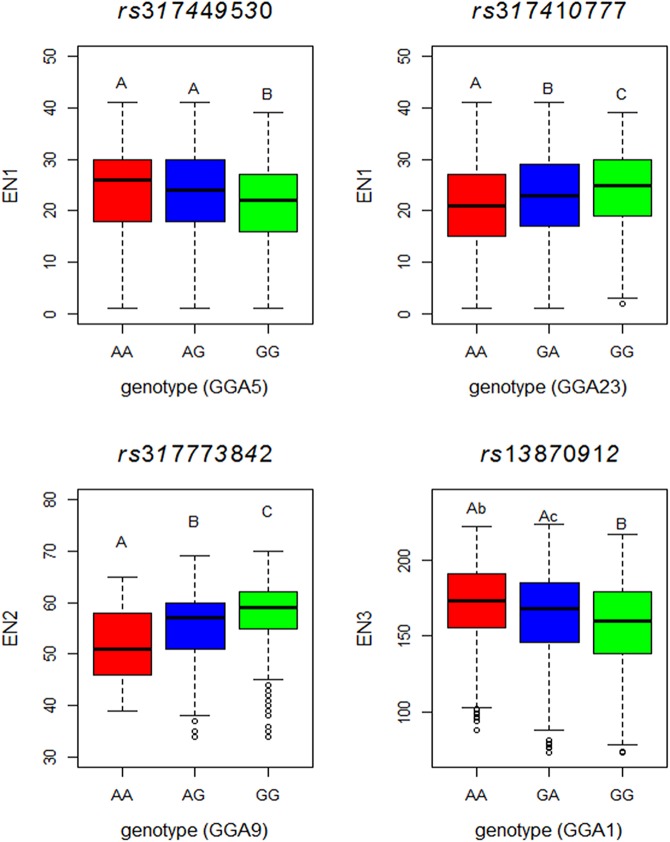
Box plot for effect of sentinel SNPs showing associations with egg number. EN1, egg numbers in pre-peak laying stage from 21 to 26 weeks of age. EN2, egg numbers in peak laying stage from 27 to 36 weeks of age. EN3, egg numbers in persistent laying stage from 37 to 72 weeks of age.

To better show the effects of genome-wide significant SNP between individuals with different genotypes, a group of box plot was shown in Fig 3. Of which the allele substitute effects of the four sentinel SNPs for EN1, EN2 and EN3 were significant (*P*-value < 0.01) between homozygous genotypes.

**Fig 4:**
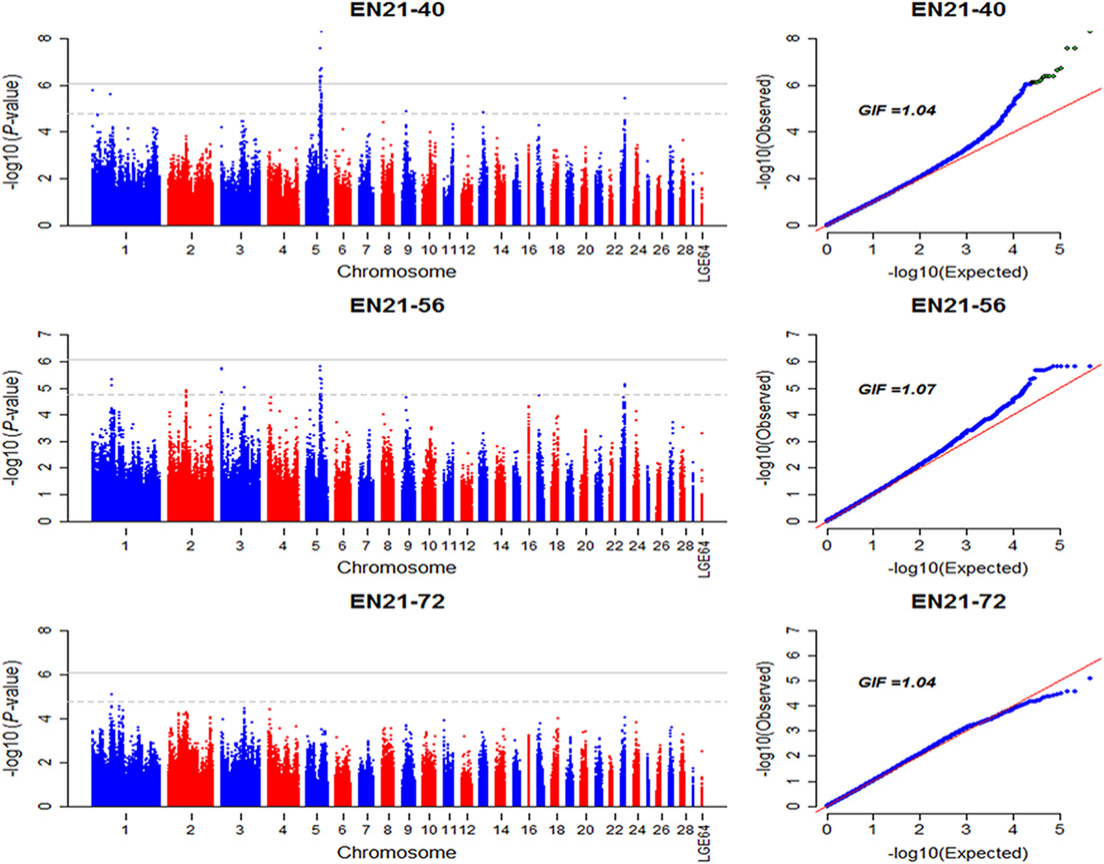
Manhattan and Q-Q plot of genome wide association study for accumulative egg number. Each dot represents a SNP in the dataset. Manhattan plot (left). EN21-40, egg numbers from 21 to 40 weeks of age. SNPs showing association with EN21-40 are mapped to one signal in chromosome 5 and a singleton in chromosome 23; EN21-56, egg numbers from 21 to 56 weeks of age. EN21-72, egg numbers from 21 to 72 weeks of age. The horizontal gray line and gray dashed line indicate the genome-wise significance threshold (*P*-value = 8.43e-7) and genome-wise suggestive significance threshold (*P*-value = 1.69e-5), respectively. GIF represents genomic inflation factor.

**Fig 5:**
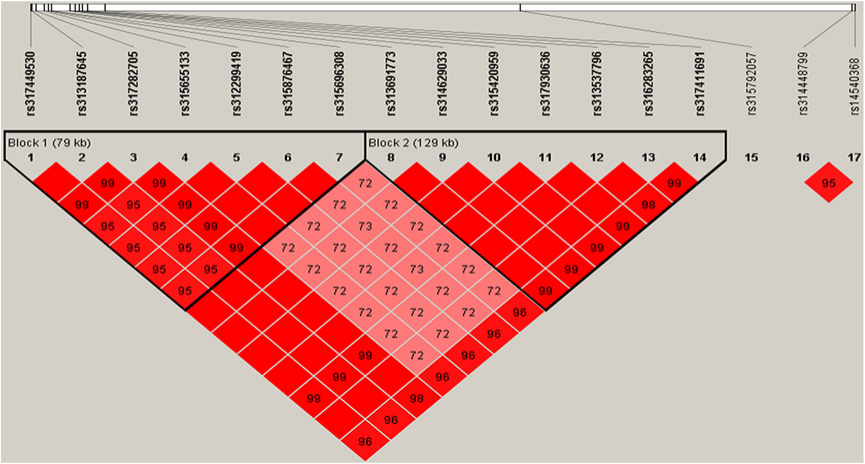
Linkage disequilibrium (r2) plot for associations with egg number betwwen 21 and 40 weeks. Solid lines mark the identified blocks.

Seventeen SNPs on GGA5 with *P*-value less than 8.43e-7 (Fig 4, Table 6) have positive effects on accumulative egg numbers from 21 to 40 weeks (EN21-40). These SNPs located on two LD blocks (Fig 5), however, the most significant SNP *rs314448799* with a candidate gene *CALM1* (*calmodulin 1 (phosphorylase kinase*, *delta)* had not fell into these two blocks. The phenotypic difference between two homozygous genotypes (CC > TT) of *this SNP* was 6.86 at a significant level (*P*-value < 0.01). Notably, loci on GGA23 and GGA9 that significantly associated with EN1 and EN2 were not significantly related to EN21-40, indicating that the genetic contributions to EN21-40 were mainly determined by significant genetic variants on GGA5 in the pre-peak laying period. As the accumulative egg number extended to 56 weeks (EN21-56), only several suggestive signals distributed on GGA1, 2, 3, 5 and 23 were detected, and the significant region on GGA5 for EN21-40 decreased to a suggestive significance for EN21-56. Moreover, one SNP on GGA1 was detected to be with a genome-wide suggestive significance for EN21-72. The difference of associated variants among EN21-40, EN21-56 and EN21-72 demonstrated that accumulative egg number was a complex trait varying with the laying age, and a proper molecular breeding program should be designed for the augment of accumulative egg number. The Manhattan and Q-Q plot for EN21-40, EN21-56 and EN21-72 are shown in Fig 4. The detailed information of markers associated with EN21-40, EN21-56 and EN21-72 is summarized in S4 Table.

**Table 6:** The information for SNPs significantly associated with egg number between 21 and 40 weeks of age.

SNP	GGA^1^	Position (bp)	Alleles^2^	MAF^3^	*P*-value	Effect ± SE^4^	Candidate/Nearest genes	Location (kb)^5^
rs314448799	5	43,152,230	C/T	0.41	5.13E-09	3.83±0.65	*CALM1*	D 8.27
rs317449530	5	40,101,576	A/G	0.31	2.53E-08	3.88±0.69	*GTF2A1*	3’ UTR
rs313187645	5	40,106,943	A/G	0.31	2.53E-08	3.88±0.69	*GTF2A1*	Intron 7
rs14540368	5	43,160,851	G/T	0.47	1.82E-07	3.39±0.65	*CALM1*	D 16.89
rs317282705	5	40,121,093	C/T	0.39	2.33E-07	3.31±0.64	*GTF2A1*	5’ UTR
rs315792057	5	41,920,622	T/C	0.41	4.19E-07	3.32±0.65	*LOC423393*	D 162.21
rs315655133	5	40,151,832	T/C	0.28	4.36E-07	3.69±0.73	*STON2*	Exon 5
rs315876467	5	40,178,369	C/T	0.28	4.36E-07	3.69±0.73	*STON2*	Intron 4
rs315696308	5	40,181,134	A/G	0.28	4.36E-07	3.69±0.73	*STON2*	Intron 4
rs312299419	5	40,167,320	G/T	0.28	4.55E-07	3.68±0.73	*STON2*	Intron 4
rs317411691	5	40,377,665	G/A	0.36	6.15E-07	3.30±0.66	*SEL1L*	U 131.90
rs313537796	5	40,293,741	A/C	0.17	7.01E-07	4.30±0.86	*SEL1L*	U 47.98
rs313691773	5	40,247,836	T/C	0.17	7.62E-07	4.30±0.86	*SEL1L*	U 2.08
rs314629033	5	40,249,984	G/T	0.17	7.62E-07	4.30±0.86	*SEL1L*	U 4.22
rs315420959	5	40,267,439	G/A	0.17	7.62E-07	4.30±0.86	*SEL1L*	U 21.68
rs317930636	5	40,284,430	C/T	0.17	7.62E-07	4.30±0.86	*SEL1L*	U 38.67
rs316283265	5	40,314,575	A/G	0.17	7.62E-07	4.30±0.86	*SEL1L*	U 68.81

^1^: Chicken chromosome.

^2^: first listed marker is minor allele.

^3^: minor allele frequency.

^4^: allele substitution effect.

^5^: D and U indicate that the SNP is upstream and downstream of the gene, respectively; UTR indicates untranslated region.

#### Egg laying rate

**Fig 6:**
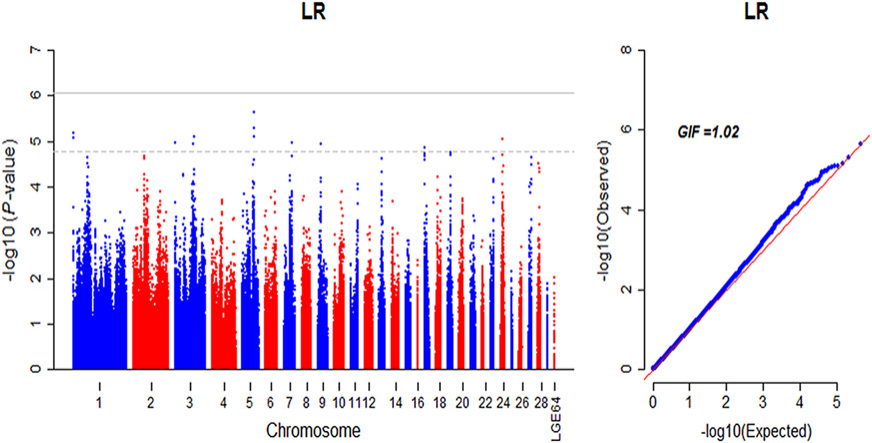
Manhattan and Q-Q plot of genome wide association study for egg laying rate. Each dot represents a SNP in the dataset. Manhattan plot (left). LR, egg laying rate between 25 and 40 weeks of age. The horizontal gray line and gray dashed line indicate the genome-wise significance threshold (P-value = 8.43e-7) and genome-wise suggestive significance threshold (P-value = 1.69e-5), respectively. GIF represents genomic inflation factor.

No genome-wide significant SNP was detected for egg laying rate (LR), but a number of suggestive significant regions were identified in various chromosomes (Fig 6), indicated that genes contributing to LR were in assorted networks regulating the laying in different weeks old. Accordingly, we used the chromosome-wide significant *P*-value, which was calculated from Bonferroni correction based on the independent tests of each autosome, as the criteria to screen associated SNPs. The identified 27 associations were located on GGA3, 5, 7, 9, 13, 17, 19, 23, 24, 27 and 28, which together explained 8.05% phenotypic variance of LR. The gene ontology analysis showed that 530 nearby genes were enriched in two significant (*P*-value < 0.05) GO terms, including lipid localization and regulation of fatty acid biosynthetic process (S5 Table). The information of associated SNPs and promising candidate genes were presented in S6 Table, respectively.

#### Age at first egg

**Fig 7:**
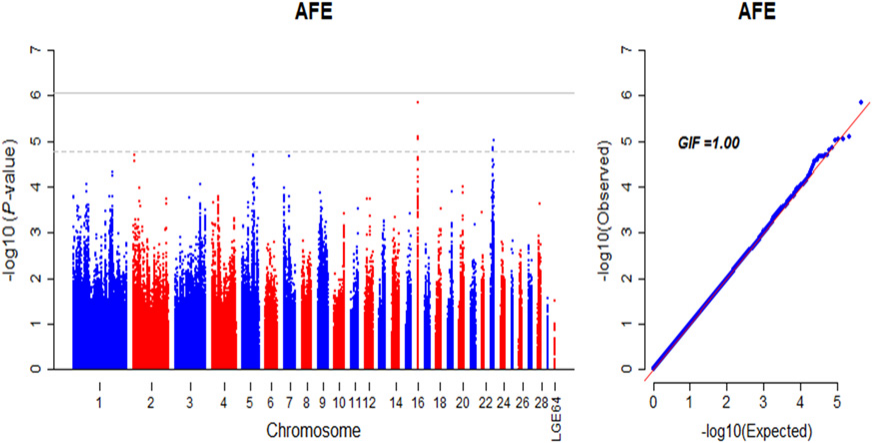
Manhattan and Q-Q plot of genome wide association study for age at first egg. Each dot represents a SNP in the dataset. Manhattan plot (left). AFE, age at first egg. The horizontal gray line and gray dashed line indicate the genome-wise significance threshold (P-value = 8.43e-7) and genome-wise suggestive significance threshold (P-value = 1.69e-5), respectively. GIF represents genomic inflation factor.

Two regions locating on GGA16 and GGA23 were detected to be associated with age at first egg (AFE) surpassing the suggestive genome-wise significance (Fig 7 and S7 Table). The most significant SNP (*rs313725141*, *P*-value = 1.36☓10^−6^) with a positive effect on AFE was in the downstream 0.75 kb of *TAP2* (*ATP-Binding Cassette*, *Sub-Family B (MDR/TAP)*) on GGA16. Some other annotated genes encompassed *MHC class IV antigen* (*B-G*), *guanine nucleotide binding protein (G protein)*, *beta polypeptide 2-like 1* (*GNB2L1*), *Transporter 2*, and *tripartite motif containing 27* (*TRIM27*) on GGA16, and *stathmin 1* (*STMN1*), *POU domain*, *class 3*, *transcription factor 1* (*POU3F1*) and *glutamate receptor*, *ionotropic*, *kainate 3* (*GRIK3*) on GGA23.

## Discussion

In the current study, we conducted a GWA analysis in an F_2_ resource population to identify the genetic variants for egg number in a more comprehensive standpoint. The purpose of F_2_ design was to generate larger genetic variation through the DNA recombination. The most associated markers are therefore expected to reside in proximity of the causal variants, and then maximizing difference of interested trait values in the population [28]. A number of GWASs have been performed on crosses between genetically and phenotypically divergent chicken lines focusing on body weight, growth, carcass traits and appearance [15, 29–31]. Our work was the first GWAS in a layer-based (WL ☓ DBS) resource population. GWA analysis was performed with genome-wide efficient mixed-model association (GEMMA) method. The algorithm is faster and exact, making GWAS computationally practical for large numbers of individuals [17]. Herein, the population stratification was adjusted using fixed and random effects, indicated that the GEMMA method was effective for population stratification and the F_2_ resource population was suitable for GWA analysis.

### Genetic parameter estimates

For the heritability estimates, pedigree-based heritability for EN1 coincided with previously reported estimates [12, 32], while heritability estimates for other four laying stages were not suitable for comparing with previous studies due to the time difference. The pedigree-based heritability estimates for EN at the late laying period (EN3-5) were smaller than the early laying period (EN1-2), agreed well with previous study that the heritability estimate decreased with age due to the increase of environmental variance [33]. Estimates of SNP-based heritability were slightly lower than those estimates from pedigree-based heritability, indicated that causal variants were well covered by the 600 K SNP arrays, and common SNPs (minor allele frequency > 0.01) had explained sufficient genetic variance for traits. However in this study, we found a higher SNP-based estimate of EN1. We inferred that the SNP information of EN1 would theoretically capture information about segregation not captured by pedigree information [34]. In addition, fewer associated SNPs are detected for EN2-5 than EN1, which may be a factor that leads to high heritability estimates of EN1. With respect to the genetic correlations among EN1-5, the pedigree-based estimates were coincided with previous estimates at a high level [12]. In current study, pedigree-based genetic correlations among EN1-5 were higher than those estimates using SNP data. The low SNP-based genetic correlations were in accord with the few numbers of shared SNPs in the GWA analyses of EN1-5. It was reported that genetic parameter estimates could be more accurate using genomic relationships, because dense genotype data permit the exploitation of small differences in the proportions of genome shared among apparently unrelated individuals [35]. Besides, genomic relationship matrix describes relationship between relatives more precisely, which reflects that actual relationship may deviate from their expectation because of Mendelian sampling [36]. Due to the analyzes of the partial genetic independence of egg number at different ages and the identification of variants for egg number at each laying period, our work provided valuable insight in the breeding program for the dynamic egg number [37]. In addition, the antagonistic relationship between AFE and egg number indicated that hens were genetically inclined to sex maturity at earlier age hence laid more eggs [38].

### Egg number

Egg number has been studied in various manners for several decades. In the current study, we analyzed the genetic architecture for egg number in five separate laying periods and three accumulative egg number traits that commonly used in the selection programs. To our knowledge, it is the first time to conduct a GWA analysis for egg number in different laying stages that derived from the overall laying period from 21 to 72 weeks. Moreover, the study of longitudinal egg number has enabled the identification of genetic variants influencing trait values over time and may be a useful tool to capture age-independent variation [39]. In the current study, four age-independent genomic regions were detected, but no consistent variation was found to be associated with egg number over the whole laying cycle. The sentinel SNP *rs317449530* for EN1 was located in 3’-UTR of *GTF2A1* gene on GGA5. *GTF2A1* is a general transcription factor that interacts with the TFIID-promoter complex required for transcription initiation by RNA polymerase II [40]. In human cancer research, it is an excellent candidate biomarker for ovarian tumor [41]. Another sentinel SNP *rs317510777* was situated nearby the *CLSPN* gene on GGA23, which encodes claspin protein involving in monitoring of DNA replication and sensoring of DNA damage in mammals [42]. Furthermore, claspin expression is significantly high in cervical cancer cell and link to human papillomavirus-related high grade lesions of uterine cervix [43]. Therefore, *GTF2A1* and *CLSPN* were supposed to be associated with the function of ovary and uterus hence influenced the egg production. The significant associated SNP on GGA9 for EN2 was located in 3’ UTR of *FARSB* gene, which encodes a highly conserved enzyme that belongs to the aminoacyl-tRNA synthetase (ARSs) family (http://www.genecards.org/cgi-bin/carddisp.pl?gene=FARSB) [44], and mutations in genes encoding ARSs lead to neurodegeneration in human [45]. The significant association for EN3 was located in the intron 18 of *KIAA1549* gene on GGA1, which has been found to be fused to the BRAF oncogene in many cases of pilocytic astrocytoma in human (http://www.genecards.org/cgi-bin/carddisp.pl?gene=KIAA1549) [44]. As the function of the gene *FARSB* and *KIAA1549* were undefined in chickens, we inferred that they interacted with central nervous system to regulate egg production based on the previous researches in human.

Regarding the accumulative egg number, the longer the laying period was, the fewer significant associations were detected for egg numbers. This indicated that the process of egg laying was intricate controlled by various factors involving genetic, physiological age, nervous, humoral regulation and environment. The SNPs that were associated with EN21-40 and EN21-56 did not coincide with previously reported QTL and SNP for similar traits [2, 4]. This discrepancy may be due to the difference of the original breeds used to construct the resource population, i.e. the reciprocal cross between WL and DBS in the present study. The potential candidate gene *CALM1* for EN21-40 on GGA5 is a prototypical calcium sensor [46]. Calcium is known to be involved in the regulation of theca cell androstenedione production and uterine contractility in laying hens [47, 48], which have the direct effect on the egg production. In a recent study, *CALM1* was found to be highly expressed in the ovary of the laying geese [49] implied that *CALM1* might be involved with the process of egg laying. With respect to the EN21-72, it was a new GWAS trait of accumulative egg number without any previous study. As no significant SNP was identified, and even regions with the genome-wide suggestive significance were few for EN21-72. Our findings suggested that accumulative egg numbers during 21 to 72 weeks of age may be more influenced by environment and marker assist selection or genomic selection for EN21-72 may be not a smart strategy in the egg production breeding.

### Egg laying rate

No significant SNP was detected in the GWA analysis of LR. However, a sum of 27 chromosome-wide significant SNPs that located on different chromosomes was identified. The 27 chromosome-wide significant SNPs accounted for 37.08% of the total phenotypic variation that explained by entire genome SNP variants (0.21, SNP-based heritability in Table 3). These indicated that LR was a complex and moderately polygenic trait, since the identified potential mutants (suggestive significance) were scattered across the entire genome with small individual effects. Although they could not reach genome-wide significance level, they together could explain considerable genetic variations [50, 51]. Moreover, in the absence of population stratification that was corrected by mixed linear mode, the deviation of several SNPs at the end of Q-Q plot indicated the presence of SNPs with small effect, and slightly inflated test statistic (1.02) also implied a polygenic nature of LR [52, 53]. The above candidate gene analysis for EN21-40 had implied that *CALM1* might be involved with the process of egg laying hence influenced the egg laying rate from 25 to 40 weeks of age. Regarding other candidate genes with unknown functions in chickens, we found that a majority of them, such as *TSN*, *THYN1*, *OPCML*, *SPPL2B* and *TCF3*, were associated with immune and cytokines, which played essential modulatory roles in the regulation of ovarian function [54].

### Age at first egg

Age at first egg (AFE) is an important production trait that influences egg number and egg weight for egg-type chicken. The previous QTL mapping studies revealed AFE related QTLs located on GGA1, 2, 3, 4, 5, 7, 11, 13, 24 and Z (http://www.animalgenome.org/cgi-bin/QTLdb/GG/). Recently, SNPs on GGA13 [4] and GGA20 [5] significantly associated with AFE. Herein, our GWAS didn’t identified genome-wide significant SNPs on autosomes and sex chromosomes (The current analysis excluded the markers on the sex chromosomes), but revealed novel suggestive genome-wise significant loci on GGA16 and GGA23. A promising candidate gene *TAP2* may responsible for the appearance of the antigen presentation pathways in the primordial MHC [55], suggested that AFE was associated with immunological process in the investigated population.

In summary, we identified nine age-independent novel loci for egg number in pre-peak, peak and persistent laying periods of chicken at genome-wide significance. Our analyses demonstrated that there exist different genetic controls for each laying period. Further functional analyses of candidate genes *GTF2A1*, *CLSPN*, *FARSB* and *KIAA1549* based on reproduction and nervous system using molecular and cell biology technology will provide a new vision to study the egg number. The identified region on GGA5 for EN21-40 was a promising QTL that could be potentially applied in marker assistant selection in breeding program to improve egg number. Moreover, *CALM1* was identified as a putative candidate gene for EN21-40 and egg laying rate, which needs further study.

## Supporting Information

S1 Fig Egg production curve of the F_2_ resource population in the laying cycle from 21 to 72 weeks of age. Each red triangle represents the laying rate in the respective week. (TIFF).
(TIF)

S2 Fig Manhattan and Q-Q plot of genome wide association study for egg number in two laying periods derived from the persistent laying period. Each dot represents a SNP in the dataset. Manhattan plot (left). EN4, egg numbers from 37 to 47 weeks of age; EN5, egg numbers from 48 to 72 weeks of age. The horizontal gray line and gray dashed line indicate the genome-wise significance threshold (*P*-value = 8.43e-7) and genome-wise suggestive significance threshold (*P*-value = 1.69e-5), respectively. GIF represents genomic inflation factor. (TIFF).
(TIF)

S1 Table The results of Gene Ontology (GO) analysis for egg production in the pre-peak laying period including genes in 0.5 Mb flanking size to SNPs with p < 1.69 × 10^−5^. (DOCX).
(DOCX)

S2 Table KEGG pathway significantly associated with egg production in the pre-peak laying period including genes in 0.5 Mb flanking size to SNPs with p < 1.69 × 10^−5^. (DOCX).
(DOCX)

S3 Table SNPs showing genome-wise significance of suggestive association (*P*-value < 1.69e-5) with egg number in five separate laying periods. ^1^: Chicken chromosome. ^2^: first listed marker is minor allele. ^3^: minor allele frequency. ^4^: allele substitution effect. ^5^: U and D indicates that the SNP is upstream and downstream of the gene; UTR indicates untranslated region. (XLSX).
(XLSX)

S4 Table SNPs with genome-wise significance of suggestive association (*P*-value < 1.69e-5) for accumulative egg number from 21 to 40, 56 and 72 weeks of age. ^1^: Chicken chromosome. ^2^: first listed marker is minor allele. ^3^: minor allele frequency. ^4^: allele substitution effect. ^5^: U and D indicates that the SNP is upstream and downstream of the gene; UTR indicates untranslated region. (XLSX).
(XLSX)

S5 Table The results of Gene Ontology (GO) analysis for egg laying rate from 25 week to 40 week including genes in 0.5 Mb flanking size to SNPs with a chromosome-wise significant *P*-value. (DOCX).
(DOCX)

S6 Table SNPs showing genome-wise significance of suggestive association (*P*-value < 1.69e-5) with egg laying rate. ^1^: Chicken chromosome. ^2^: first listed marker is minor allele. ^3^: minor allele frequency. ^4^: allele substitution effect. ^5^: U and D indicates that the SNP is upstream and downstream of the gene. (XLSX).
(XLSX)

S7 Table SNPs showing genome-wise significance of suggestive association (*P*-value < 1.69e-5) with age at first egg. ^1^: Chicken chromosome. ^2^: first listed marker is minor allele. ^3^: minor allele frequency. ^4^: allele substitution effect. ^5^: U and D indicates that the SNP is upstream and downstream of the gene. (XLSX).
(XLSX)
