# Supplementary figures and images for: Identification of Promising Mutants Associated with Egg Production Traits Revealed by Genome-Wide Association Study
Source: PLoS One. 2015 Oct 23;10(10):e0140615. doi: 10.1371/journal.pone.0140615 (PMC4619706; doi:10.1371/journal.pone.0140615)

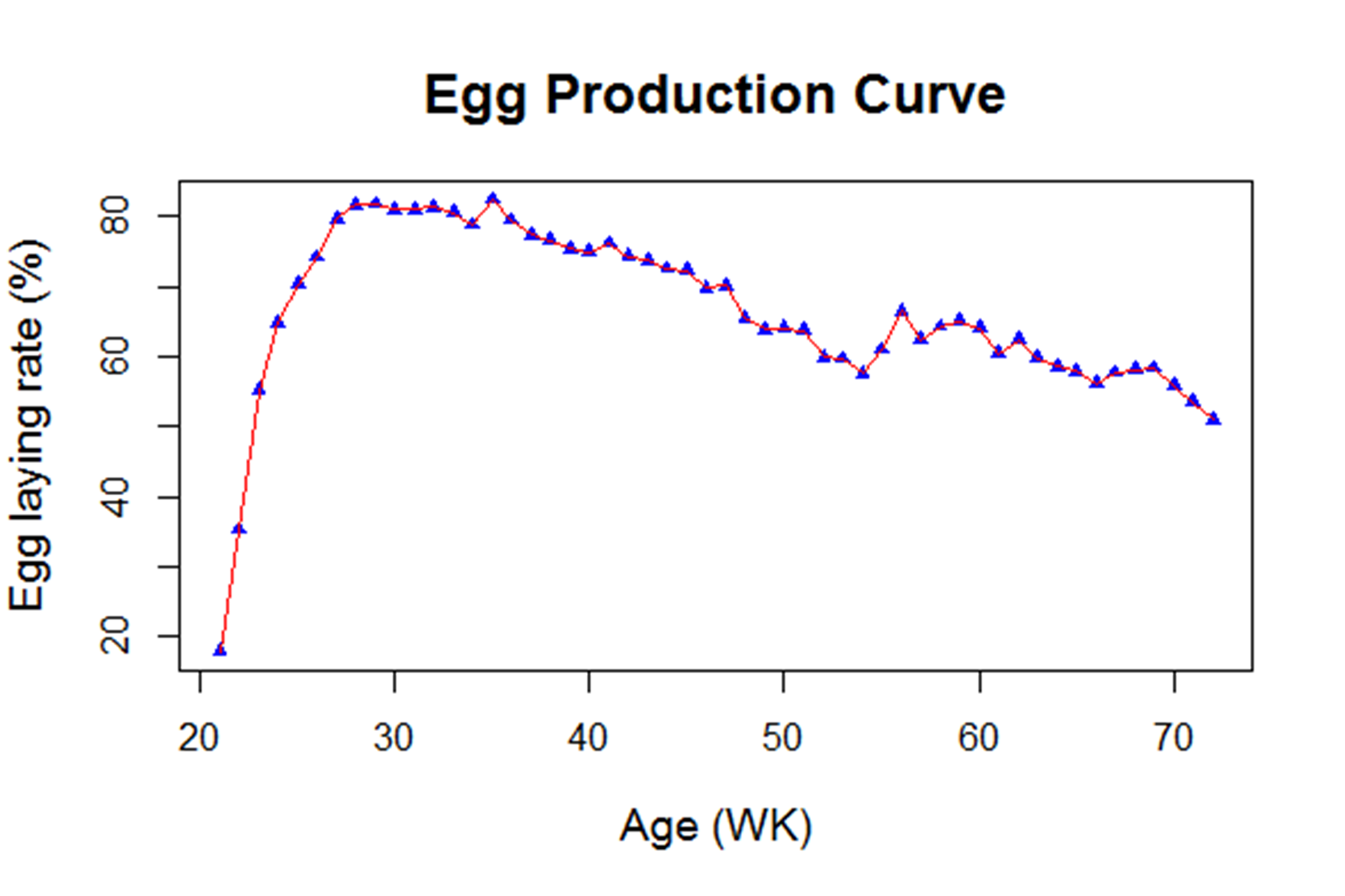

Supplement: S1 Fig — Each red triangle represents the laying rate in the respective week. (TIFF). (TIF) [file pone.0140615.s001.tif]

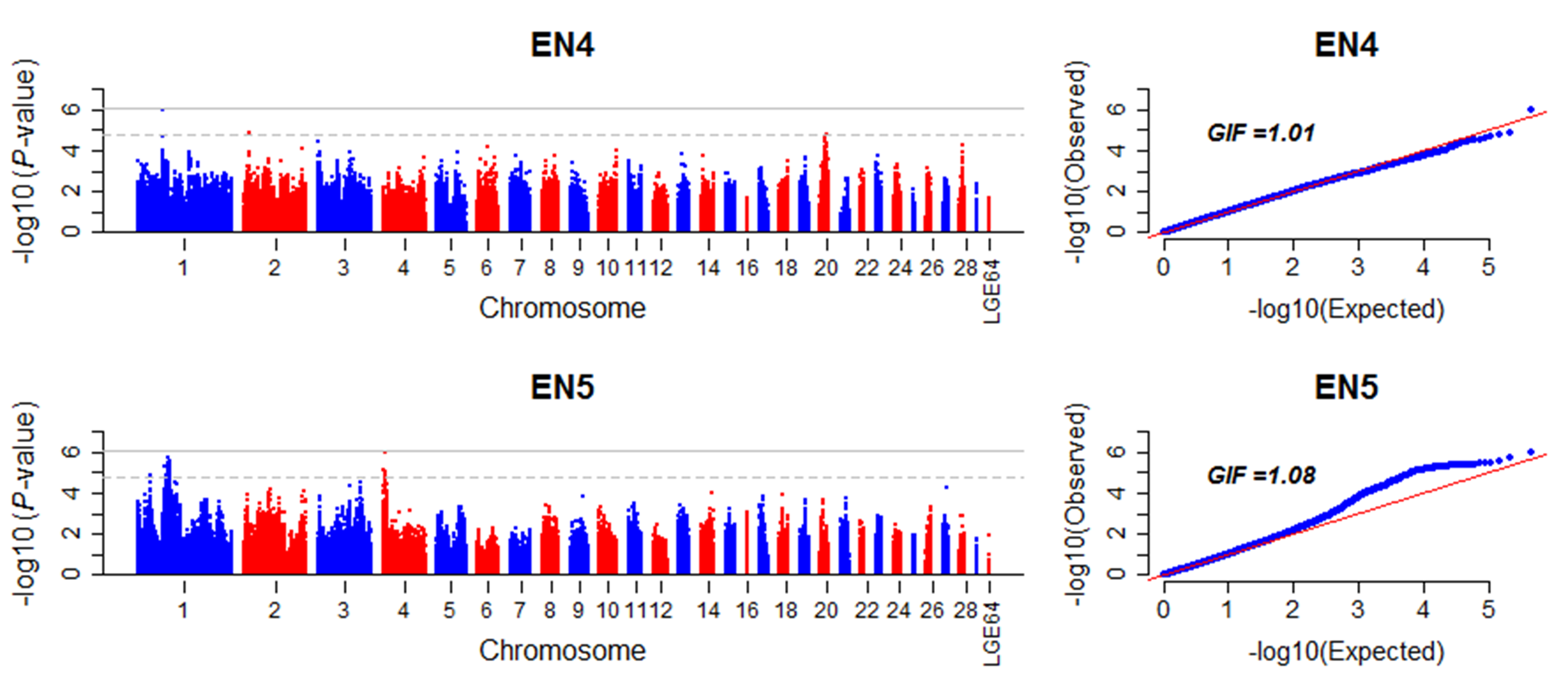

Supplement: S2 Fig — Each dot represents a SNP in the dataset. Manhattan plot (left). EN4, egg numbers from 37 to 47 weeks of age; EN5, egg numbers from 48 to 72 weeks of age. The horizontal gray line and gray dashed line indicate the genome-wise significance threshold (P-value = 8.43e-7) and genome-wise suggestive significance threshold (P-value = 1.69e-5), respectively. GIF represents genomic inflation factor. (TIFF). (TIF) [file pone.0140615.s002.tif]
