# Supplementary material for: Identification of Promising Mutants Associated with Egg Production Traits Revealed by Genome-Wide Association Study
Source: PLoS One. 2015 Oct 23;10(10):e0140615. doi: 10.1371/journal.pone.0140615 (PMC4619706; doi:10.1371/journal.pone.0140615)
Supplement: S1 Table — (DOCX). (DOCX) [file pone.0140615.s003.docx]

**Table S1. The results of Gene Ontology (GO) analysis for egg number in the pre-peak laying period including genes in 0.5 Mb flanking size to SNPs with p < 1.69 × 10^-5^.**

| **GO sub-ontology** | **GO term** | **Go term description** | **David *P*-value** | **Involved genes** |
| --- | --- | --- | --- | --- |
| Biological process | GO:0019882 | antigen processing and presentation | 2.24E-16 | BLB2, LOC430600, CD1C, CD1B, TAPBP, LOC417058, LOC417057, PROCR, LOC417083, TAP2, BF2, BF1, MR1, LOC417056 |
| Biological process | GO:0006955 | immune response | 8.75E-06 | BLB2, LOC417058, LOC417057, PROCR, LOC417083, LOC430600, CD1C, CD1B, BF2, BF1, MR1, LOC417056 |
| Biological process | GO:0006468 | protein amino acid phosphorylation | 0.00312 | PDK1, ZAK, RYK, PASK, MYO3B, MAPK11, EPHA10, RPS6KA5, PDIK1L, MAPK12, STK40, PAK2, TLK1 |
| Biological process | GO:0048002 | antigen processing and presentation of peptide antigen | 0.004396 | TAP2, BF2, TAPBP |
| Biological process | GO:0016310 | phosphorylation | 0.010696 | PDK1, ZAK, RYK, PASK, MYO3B, MAPK11, EPHA10, RPS6KA5, PDIK1L, MAPK12, STK40, PAK2, TLK1 |
| Biological process | GO:0016570 | histone modification | 0.011786 | RPS6KA5, ING5, MEAF6, HAT1 |
| Biological process | GO:0016569 | covalent chromatin modification | 0.013609 | RPS6KA5, ING5, MEAF6, HAT1 |
| Biological process | GO:0006473 | protein amino acid acetylation | 0.015548 | ING5, MEAF6, HAT1 |
| Biological process | GO:0016573 | histone acetylation | 0.015548 | ING5, MEAF6, HAT1 |
| Biological process | GO:0006796 | phosphate metabolic process | 0.016261 | PDK1, ZAK, RYK, MYO3B, PASK, MAPK11, EPHA10, PTPRU, RPS6KA5, PDIK1L, MAPK12, STK40, PAK2, TLK1 |
| Biological process | GO:0006793 | phosphorus metabolic process | 0.016261 | PDK1, ZAK, RYK, MYO3B, PASK, MAPK11, EPHA10, PTPRU, RPS6KA5, PDIK1L, MAPK12, STK40, PAK2, TLK1 |
| Biological process | GO:0043543 | protein amino acid acylation | 0.019805 | ING5, MEAF6, HAT1 |
