# Supplementary material for: Identification of Promising Mutants Associated with Egg Production Traits Revealed by Genome-Wide Association Study
Source: PLoS One. 2015 Oct 23;10(10):e0140615. doi: 10.1371/journal.pone.0140615 (PMC4619706; doi:10.1371/journal.pone.0140615)
Supplement: S2 Table — (DOCX). (DOCX) [file pone.0140615.s004.docx]

**Table S2. KEGG pathway significantly associated with egg production in the pre-peak laying period including genes in 0.5 Mb flanking size to SNPs with p < 1.69 × 10^-5^.**

| **KEGG entry** | **Pathway name** | **David *P*-value** | **Involved genes** |
| --- | --- | --- | --- |
| gga04514 | Cell adhesion molecules (CAMs) | <0.001 | BLB2, CLDN18, LOC417057, ITGA6, NRXN3, LOC430600, BF2, BF1, MR1 |
| gga04010 | MAPK signaling pathway | 0.035 | RPS6KA5, MAPK12, PAK2, ZAK, SOS2, MAPK11, STMN1 |
