# Supplementary material for: Identification of Promising Mutants Associated with Egg Production Traits Revealed by Genome-Wide Association Study
Source: PLoS One. 2015 Oct 23;10(10):e0140615. doi: 10.1371/journal.pone.0140615 (PMC4619706; doi:10.1371/journal.pone.0140615)
Supplement: S5 Table — (DOCX). (DOCX) [file pone.0140615.s007.docx]

**Table S5. The results of Gene Ontology (GO) analysis for egg laying rate from 25 to 40 week including genes in 0.5 Mb flanking size to SNPs with a chromosome-wise significant *P*-value.**

| **GO sub-ontology** | **GO term** | **Go term description** | **David *P*-value** | **Involved genes** |
| --- | --- | --- | --- | --- |
| Biological process | GO:0010876 | lipid localization | 0.02 | APOA4, APOA1, STAT5B, APOA5, ATP8B3 |
| Biological process | GO:0042304 | regulation of fatty acid biosynthetic process | 0.04 | APOA4, BRCA1 |
